# Cost-effectiveness analysis of toripalimab plus chemotherapy as the first-line treatment in patients with advanced non-small cell lung cancer (NSCLC) without EGFR or ALK driver mutations from the Chinese perspective

**DOI:** 10.3389/fphar.2023.1133085

**Published:** 2023-05-17

**Authors:** Kexun Zhou, Pei Shu, Hanrui Zheng, Qiu Li

**Affiliations:** ^1^ Department of Medical Oncology, Cancer Center, West China Hospital, Sichuan University, Chengdu, China; ^2^ Department of Pharmacy, West China Hospital, Sichuan University, Chengdu, China

**Keywords:** cost-effectiveness, toripalimab, chemotherapy, non-small cell lung cancer, Chinese payer’s perspective

## Abstract

**Objectives:** The results of a CHOICE-1 study demonstrated the superior efficacy of toripalimab (anti-PD-1 antibody) plus chemotherapy for patients with advanced non–small cell lung cancer (NSCLC), with a manageable safety profile. This study was performed to evaluate the economic value of this treatment for this patient population from the Chinese payer’s perspective.

**Materials and methods:** Basic data were derived from the CHOICE-1 study. Markov models were developed to simulate the process of advanced NSCLC, including the progression-free survival (PFS), progressive disease (PD), and death in intention-to-treat (ITT) populations, as well as patients with squamous or non-squamous NSCLC. The cost was obtained from the local institution, and the value of utilities referred to previous studies. Monte Carlo simulations were performed to depict the probabilistic scatter plots of the incremental cost-effectiveness ratio (ICER) and acceptability curves, aiming to address the uncertainty of model inputs.

**Results:** Compared with standard chemotherapy, toripalimab plus chemotherapy yields an ICER of $21,563 per quality-adjusted life year (QALY) in the ITT population. For patients with squamous NSCLC, comparing the combined therapy with chemotherapy led to an ICER of $18,369 per QALY, while the ICER was $24,754 per QALY in patients with non-squamous NSCLC. With the threshold of willingness to pay we set ($37,653 per QALY), toripalimab plus chemotherapy was cost-effective in these patient populations.

**Conclusion:** For patients with advanced NSCLC, toripalimab plus chemotherapy was an optimal choice as first-line treatment, regardless of histology.

## 1 Introduction

Worldwide, lung cancer ranks as the second most frequent cancer and the leading cause of cancer death, with nearly 2.2 million newly diagnosed cases and 1.8 million deaths reported in 2020 (Sung et al., 2021). Among them, cases from China accounted for nearly 30% (Qiu et al., 2021). Lung cancer can be broadly classified into two classical types, non-small cell lung cancer (NSCLC) and small cell lung cancer (SCLC), and NSCLC represents almost 85% of the cases (Rodriguez-Lara et al., 2018).

With the development of cancer treatments, significant efforts have been made to improve the outcomes of patients with lung cancer. Targeted therapies are optimal for advanced NSCLC with gene mutations, while for patients without driver mutations, immunotherapies alone or with traditional chemotherapies have led to a paradigm shift in the treatments (Reck et al., 2022). Notably, immunotherapies have yielded different responses in NSCLC as the efficacy depended on various factors (e.g., tumor mutational burden (TMB) and the expression of PD-L1). Such disparities have attracted widespread attention, aiming to find more accurate predictive biomarkers.

Toripalimab is a novel anti-PD-1 antibody that engages a differential domain on PD-1 than existing anti-PD-1 antibodies (Liu et al., 2019). Toripalimab monotherapy has shown promising antitumor effects with tolerable safety among patients with advanced NSCLC in a phase I trial (Wang et al., 2020). Based on this, a subsequent phase III trial (CHOICE-01) was conducted to evaluate whether toripalimab plus chemotherapy could provide better survival outcomes than chemotherapy in the first-line treatment of advanced NSCLC without EGFR/ALK driver mutations. Enrolled patients came from medical centers across China. The results indicated the combined therapy significantly improved the progression-free survival (PFS) and overall survival (OS) compared to its competitor (median PFS, 8.4 vs. 5.6 months; hazard ratio (HR) = 0.49; *p* < 0.0001; median OS, not reached vs. 17.1 months; HR = 0.69; *p* = 0.0099). Safety profiles were in line with a previous study (Wang et al., 2022). Based on these results, the protocol was recently approved by the Chinese National Medical Products Administration as a first-line treatment for this patient population.

Meanwhile, existing evidence indicated that the Chinese economic burden of lung cancer was $25,029 million in 2017, with an increasing trend in the next decade (Liu et al., 2021). With the growth of the field of immunotherapy, increasing attention has been focused on its economic value. Higher medical costs have usually decimated the generalizability of immune checkpoint inhibitors. Here, we conducted a cost-effectiveness analysis based on the results of CHOICE-01 to compare the economic value of toripalimab plus chemotherapy with chemotherapy alone from the Chinese payer’s perspective.

## 2 Methods

### 2.1 Model structure

**FIGURE 1:**
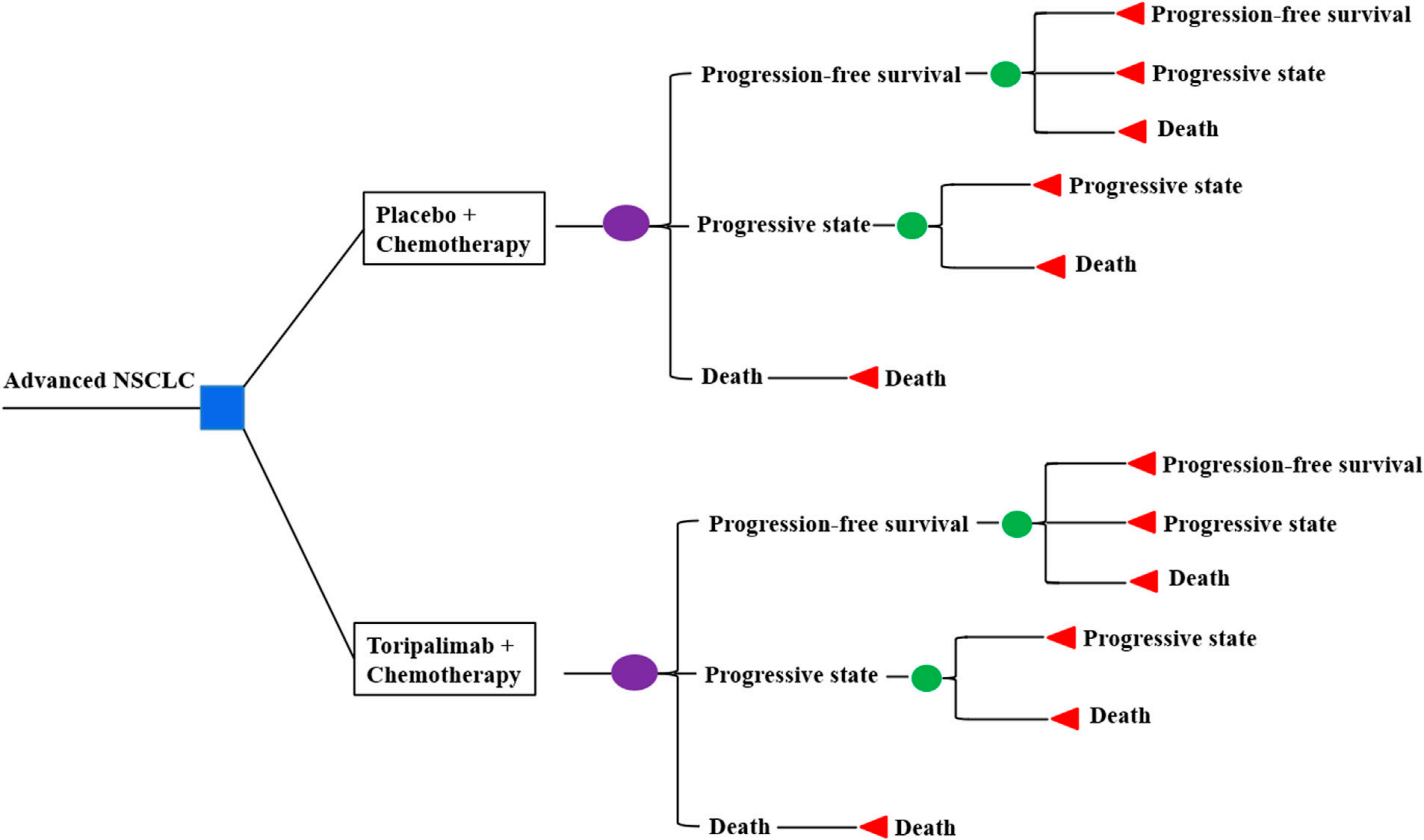
Markov models.

Markov models were constructed with clinical data derived from the CHOICE-01 study. We compare cost and quality-adjusted life years (QALYs) in three patient populations: a) intention-to-treat (ITT) population, b) patients with squamous NSCLC, and c) patients with non-squamous NSCLC. Three health states were considered: progression-free survival (PFS), progressive disease (PD), and death (Figure 1). Three weeks was set as a cycle length, while a time horizon of 10 years was applied coupled with half-cycle correction. Costs of the drug, hospitalization, management of adverse events (AEs), and treatments for the PD state were included, as well as the administration of monitoring. Prices were derived from the West China Hospital and converted into US dollars ($1 = RMB 7.2363, 28 Oct 2022). TreeAge Pro 2022 and R software (version 3.5.2) were applied to perform the analyses.

### 2.2 Clinical inputs

Patients were randomized to receive toripalimab with chemotherapy or placebo with chemotherapy. Toripalimab or placebo was administered at 240 mg every 3 weeks. Chemotherapies for patients with non-squamous NSCLC were pemetrexed (500 mg/m^2^) plus cisplatin (75 mg/m^2^) or carboplatin (AUC = 5 mg/mL/min) every 3 weeks for 4-6 cycles, followed by pemetrexed plus toripalimab or placebo maintenance. Chemotherapies for patients with squamous NSCLC included nab-paclitaxel (100 mg/m^2^) on days 1, 8, and 15 once, plus carboplatin (AUC = 5 mg/mL/min) every 3 weeks for 4–6 cycles. The maintenance treatment was toripalimab or placebo. The incidences of grade ≥3 AEs were similar in the two groups. After the progression, the majority received subsequent systemic therapies. More than 50% of patients in the chemotherapy group crossed over to toripalimab. Multiple strategies were administered to patients in the combined group. Nearly half of the patients were administered immunotherapies, and sintilimab accounted for the largest proportion. Although a considerable proportion of patients received cytotoxic therapy and tyrosine kinase inhibitors (TKIs), specific details were not available. Thus, we assumed patients receiving sintilimab monotherapy were in the PD state. Relative costs are listed in Table 1.

**TABLE 1:** Base-case cost and utilities.

Parameter	Value	Range	Distribution
Cost per cycle ($)			
Toripalimab	342	239–445	Gamma
Chemotherapy			
ITT	666	466–866	Gamma
Squamous	313	220–408	Gamma
Non-S	1,018	713–1,325	Gamma
Test			
Placebo plus chemotherapy	420	294–546	Gamma
Toripalimab plus chemotherapy	439	307–571	Gamma
Treatment for PD state			
Placebo plus chemotherapy	342	239–445	Gamma
Toripalimab plus chemotherapy	299	209–389	Gamma
AEs			
Placebo plus chemotherapy	138	97–179	Gamma
Toripalimab plus chemotherapy	140	98–182	Gamma
Hospitalization	50	35–65	Gamma
Utility score			
PFS	0.673	0.47–0.87	Beta
PD	0.473	0.33–0.61	Beta

ITT, intention-to-treat population; Non-S, non-squamous; PFS, progression-free survival; PD, progressive disease; AEs, adverse events.

**FIGURE 2:**
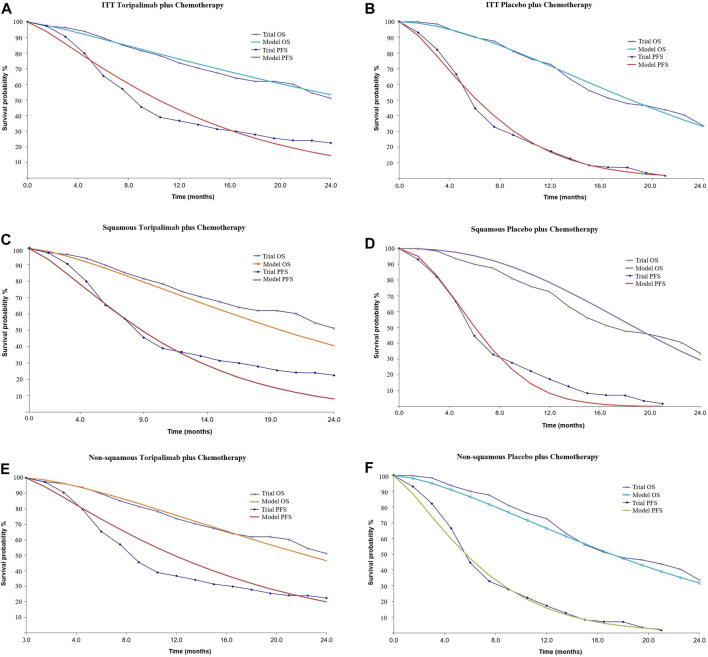
Calibrated survival curves. **(A, B)**: Intention-to-treat population, **(C, D)**: patients with squamous NSCLC, and **(E, F)**: patients with non-squamous NSCLC. ITT, intention-to-treat population; OS, overall survival; PFS, progression-free survival.

Individual data were extracted from the Kaplan–Meier survival curves in the CHOICE-01 study using Plot Digitizer software (version 2.6.8). Weibull survival models developed by Hoyle and Henley (2011) were constructed to fit the survival curves (Table 2). Further validation was performed by R software (version 3.5.2). The calibrated curves are shown in Figure 2.

**TABLE 2:** Parameters of survival distribution.

Parameter	Placebo + chemotherapy			Toripalimab + chemotherapy				
	ITT	Squamous	Non-S	ITT		Squamous		Non-S
Weibull OS model								
Intercept	3.119	3.090	3.084	3.5639		3.230		3.758
Log (scale)	−0.539	−0.851	−0.399	−0.186		−0.308		−0.175
Gamma	1.715	2.341	1.490	1.205		1.360		1.191
Lambda	0.005	0.001	0.010	0.014		0.012		0.011
Weibull PFS model								
Intercept	2.072	1.995	2.139	2.636		2.463		2.777
Log (scale)	−0.352	−0.621	−0.217	−0.203		−0.263		−0.174
Gamma	1.422	1.860	1.242	1.226		1.300		1.190
Lambda	0.053	0.024	0.070	0.040		0.041		0.037

ITT, intention-to-treat population; Non-s, non-squamous; OS, overall survival; PFS, progression-free survival; AEs, adverse events; PD, progressive disease.

### 2.3 Health outcomes

Because the quality of life was not measured in the CHOICE-01 study, we cited the utility scores from the published data (Nafees et al., 2008). Because the incidence of AEs was similar between the two groups, it was reasonable to assume that the utility scores of each state could be applied to the two groups. As a result, 0.673 was set for PFS (uPFS), 0.473 for PD (uPD), and 0 for death (uDeath).

### 2.4 Model analysis

The incremental cost-effectiveness ratio (ICER) was calculated based on the quality-adjusted life years (QALYs) gained and overall cost. Cost and QALY were discounted at an annual rate of 3%. Three times the gross domestic product (GDP) *per capita* was set as the threshold of willingness to pay (WTP) ($37,653/QALY, 2021). One-way sensitivity analysis was performed to explore the potential influence of different parameters, and variables varied across a range of ±30%. Probabilistic sensitivity analysis was performed using a Monte Carlo simulation of 1,000 iterations. A cost-effectiveness acceptability curve was depicted to reflect the probability that a treatment would be cost effective.

## 3 Results

The results of the base case analysis are shown in Table 3. Generally, toripalimab plus chemotherapy generated better QALYs but higher cost in the three patient populations. For the ITT population, compared with chemotherapy alone, combined therapy was estimated to increase costs by $14,447 with a gain of 0.67 QALYs (1.61 vs. 0.94 QALYs), leading to an ICER of $21,563 per QALY. In patients with squamous NSCLC, combined therapy yielded an ICER of $18,369 per QALY compared with the competitor. For patients with non-squamous NSCLC, toripalimab plus chemotherapy cost $16,585 more than chemotherapy alone, resulting in an ICER of $24,754 per QALY. With the threshold of WTP we set, toripalimab plus chemotherapy was cost-effective in these patient populations, regardless of histology.

**TABLE 3:** Cost-effectiveness analysis.

Parameter	Placebo + chemotherapy	Toripalimab + chemotherapy
Cost ($)		
ITT		
PFS state	9,315	21,911
PD state	4,630	6,481
Total cost	13,945	28,392
Incremental cost		14,447
Squamous		
PFS state	6,737	17,193
PD state	4,630	6,481
Total cost	11,367	23,674
Incremental cost		12,307
Non-S		
PFS state	11,893	26,627
PD state	4,630	6,481
Total cost	16,523	33,108
Incremental cost		16,585
Effectiveness (QALYs)		
PFS state	0.41	0.75
PD state	0.53	0.86
Total effectiveness	0.94	1.61
Incremental effectiveness		0.67
ICER ($/QALY)		
ITT		21,563
Squamous		18,369
Non-S		24,754

ITT, intention-to-treat population; Non-S, non-squamous; PFS, progression-free state; PD, progressive disease; QALYs, quality adjusted-life years; ICER, incremental cost-effectiveness ratio.

**FIGURE 3:**
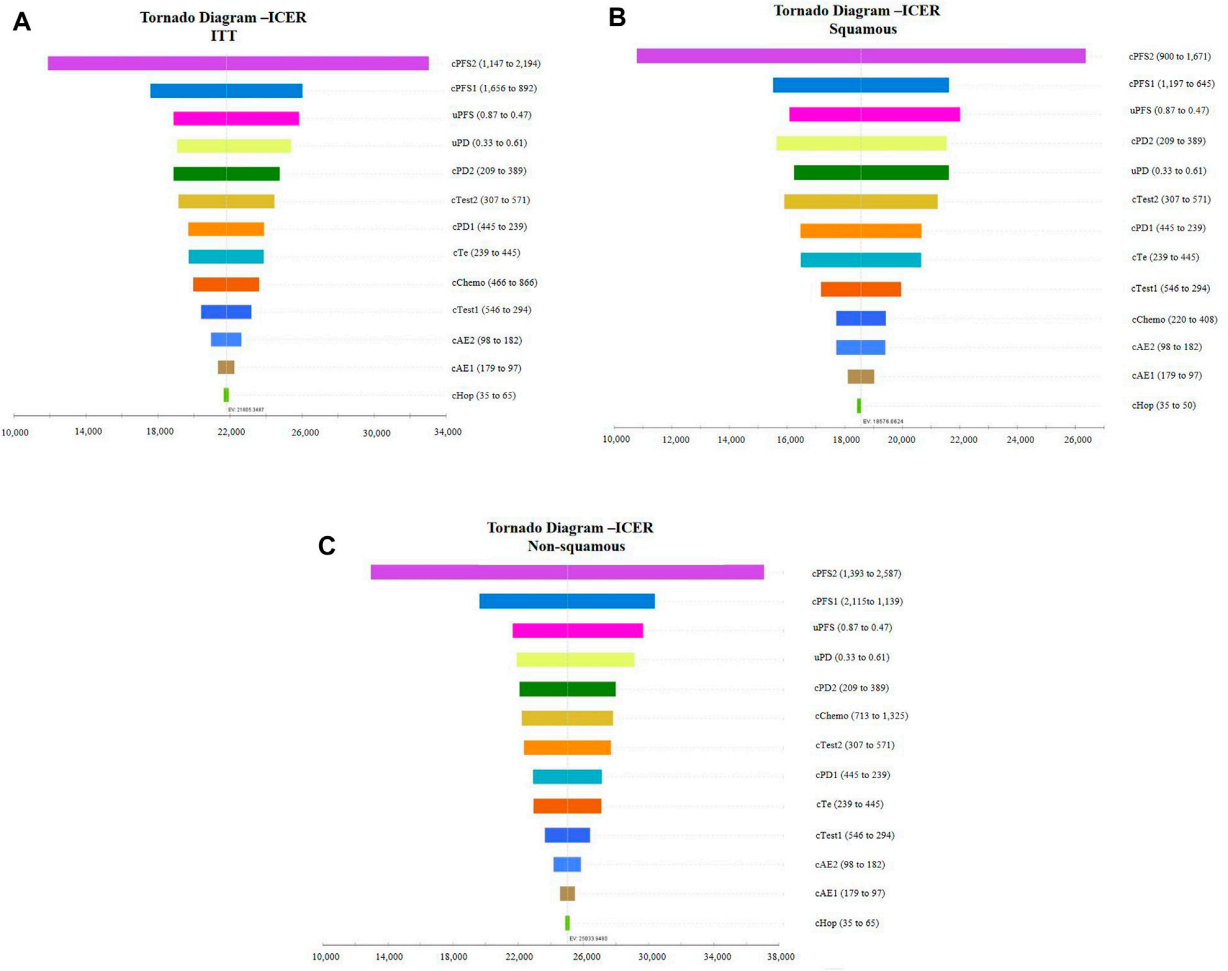
Tornado diagram of the one-way sensitivity analysis. **(A)**: Intention-to-treat population, **(B)**: patients with squamous NSCLC, and **(C)**: patients with non-squamous NSCLC. ICER, incremental cost-effectiveness ratio; ITT, intention-to-treat population.

Tornado diagrams of one-way sensitivity analyses are shown in Figure 3. The results suggested that the cost of combined treatment has the greatest influence on the ICER, regardless of histology. Following that, the cost of chemotherapy in the PFS state was the second-most sensitive parameter in the model. In the ITT population and non-squamous NSCLC base case analyses, uPFS and uPD also had a considerable impact on the ICER. For patients in the squamous NSCLC population, the cost of PD state in the combined group (cPD2) ranked as the fourth-most sensitive parameter, followed by uPD.

**FIGURE 4:**
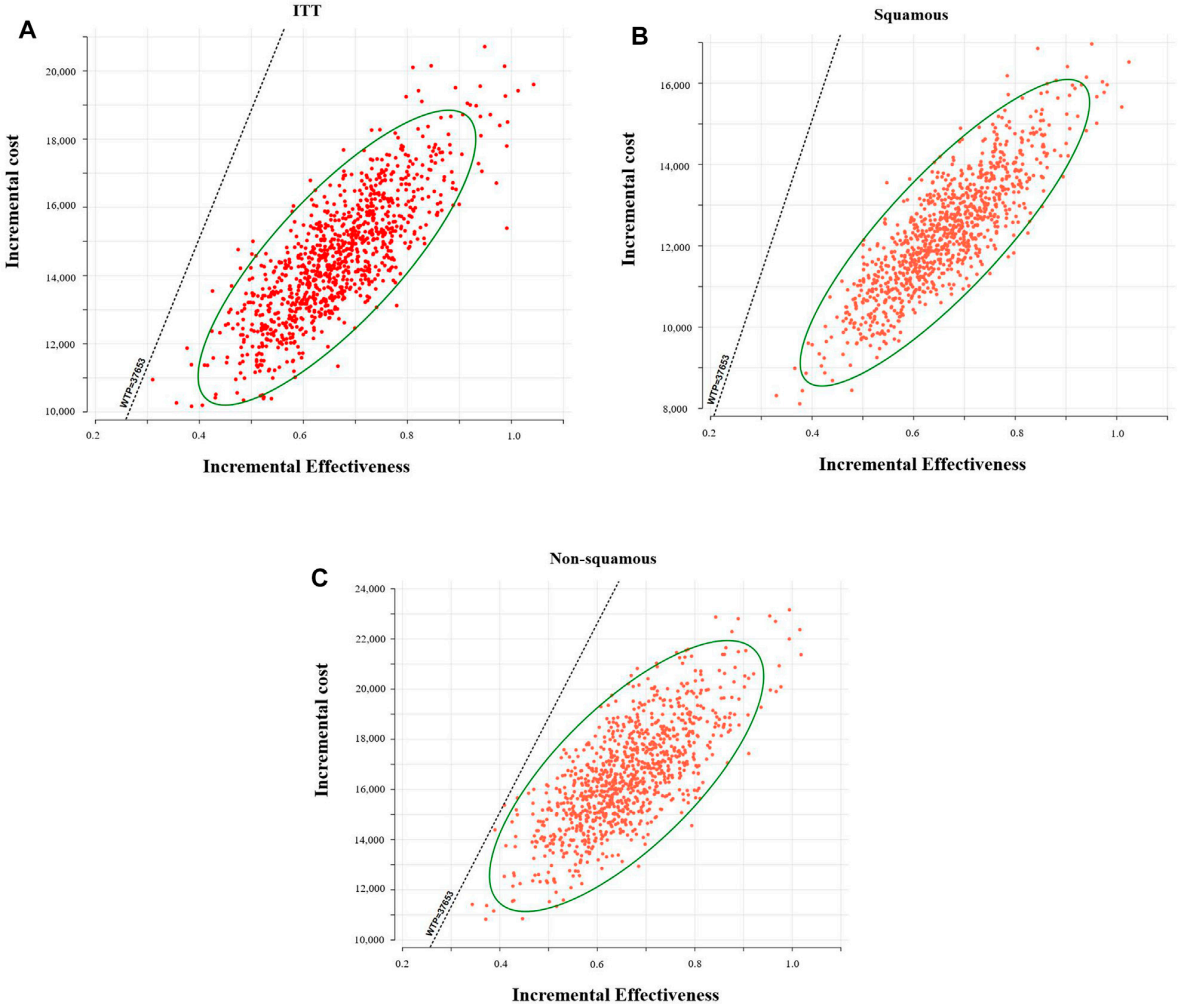
Probabilistic scatter plots of the ICER. **(A)**: Intention-to-treat population, **(B)**: patients with squamous NSCLC, and **(C)**: patients with non-squamous NSCLC. WTP, willingness to pay.

The scatter plot indicated, with the threshold of WTP we set, 100% of these ICERs (baseline, placebo plus chemotherapy; comparator, toripalimab plus chemotherapy) were located in the low right-hand quadrant (Figure 4). The acceptability curves shown in Supplementary Figure S1 also indicated that with the threshold of WTP we set, toripalimab plus chemotherapy was cost-effective for 100% of iterations.

## 4 Discussion

Based on the inspiring results of CHOICE-01, this study was conducted to compare the cost-effectiveness of toripalimab plus chemotherapy with chemotherapy alone for patients with advanced NSCLC without an EGFR/ALK mutation from the Chinese payer’s perspective. The results showed that the combined therapy cost $14,447, $12,307, and $16,585 more than the competitor and provided an additional 0.67 QALYs, leading to an ICER of $21,563/QALY, $18,369/QALY, and $24,754/QALY in the ITT, squamous NSCLC, and non-squamous NSCLC populations, respectively. Based on the threshold of WTP we set, our study indicated that the combined therapy was cost-effective for patients with advanced NSCLC, regardless of histology.

The last decade has witnessed the development of immunotherapy in advanced NSCLC, especially represented by pembrolizumab, nivolumab, atezolizumab, and ipilimumab. The success of immunotherapy in heavily pretreated, advanced NSCLC paved the way for its application as a first-line treatment (Reck et al., 2022). Despite the superior efficacy provided by immunotherapy, the results of relative cost-effectiveness analyses were not inspiring. We have performed a study to assess the cost-effectiveness of pembrolizumab monotherapy compared with traditional chemotherapy as a first-line treatment for advanced NSCLC (Zhou et al., 2019). The ICERs yielded by pembrolizumab among patients with different tumor proportion scores (TPS) were beyond the threshold of WTP at that time, which indicated that pembrolizumab was not preferred over the standard chemotherapy. Similar findings have been reported in other studies (Wan et al., 2020; Wu and Lu, 2020; Jiang and Wang, 2022). Notably, a patient assistance program (PAP) for pembrolizumab was applied in China. Briefly, patients pay for the first two cycles of pembrolizumab and receive the next two cycles for free. When the patients are eligible to continue the treatment of pembrolizumab, they pay for the fifth and sixth cycles of pembrolizumab, and the next cycles for up to 2 years are free. However, even in this context, pembrolizumab plus chemotherapy as the first-line treatment for NSCLC was still not preferred in China (Cai et al., 2021). Taken together, pembrolizumab with or without chemotherapy showed no cost-effectiveness as a front-line treatment for advanced NSCLC. Previous studies using nivolumab and atezolizumab also indicated that immunotherapy-based therapies were unlikely to be cost-effective compared with chemotherapy (Lin et al., 2020; Liu et al., 2020; Hao et al., 2021; Yang et al., 2021).

In recent years, research into PD-1/PD-L1 inhibitors has received strong interest in China. Surprisingly, inhibitors developed in China (e.g., camrelizumab, sintilimab and tislelizumab) showed non-inferior efficacy compared with imported drugs. In this case, the price of the drug becomes the key to the accessibility of the new drugs. Existing evidence indicated that inhibitors developed in China were cost-effective for advanced NSCLC. For example, Zhu et al. (2021) performed a study to investigate whether camrelizumab plus chemotherapy was cost effective in the first-line treatment for patients with metastatic non-squamous NSCLC. The results indicated that the combined therapy provided better QALYs with a much lower cost, yielding an ICER of $−7382.72/QALY. Meanwhile, another study compared the economic value of sintilimab plus chemotherapy with pembrolizumab plus chemotherapy for locally advanced or metastatic squamous NSCLC from the perspective of the Chinese health system. Owing to the price advantage, a sintilimab-based strategy was considered the optimal choice rather than the pembrolizumab-based treatment (Chen et al., 2022).

Toripalimab was the first domestic PD-1 inhibitor approved by the Chinese National Medical Products Administration. It has been widely applied for advanced melanoma, nasopharyngeal carcinoma, urothelial carcinoma, and esophageal squamous cell carcinoma. Limited evidence suggested a toripalimab-based strategy was cost-effective for patients with advanced nasopharyngeal carcinoma compared with its competitor (Tian et al., 2022; Zhu et al., 2022). A positive conclusion was also drawn in our study. Notably, we constructed models for an ITT population and patients with different histologies. Although toripalimab plus chemotherapy was the preferred choice for all enrolled patient populations, patients with squamous NSCLC benefit most from the combined treatment.

The conclusion of cost-effectiveness analysis heavily depends on the threshold of WTP, which varies in different countries. According to the guidelines of the World Health Organization (WHO), three times the GDP *per capita* is usually set as the threshold. In the current study, we found that when even twice the GDP *per capita* was set as the threshold ($25,100/QALY), toripalimab plus chemotherapy was still cost-effective. Kazibwe et al. (2022) pointed out that setting three times the GDP *per capita* as the threshold could lead to a higher probability of study interventions being cost-effective. However, this might lead policymakers to promote interventions that may not be affordable. This concern needs further validation.

This study was not without limitations. First, survival data in the model were retrospectively derived from the CHOICE-1 study, and we assumed patients receive a specific management as subsequent treatment. This assumption may create a gap with patient-level data in the real world and shift the clinical efficacy. Second, base-case cost was obtained from a local institution. Although drug prices are approximately the same in most Chinese medical centers, some differences may exist between different hospitals or regions. This limitation may affect the generalizability of our research. Third, sensitivity analyses suggested that utility scores had a substantial impact on the ICER. As the quality of life was not measured in the CHOICE study, the utility values referred to previous studies. However, we varied the utility with a range of ±30%, which could diminish the uncertainty of the models. In addition, the CHOICE-1 study further investigated whether a genomic panel was associated with the response rate (e.g., TMB, PD-L1 expression, gene mutations). The results indicated that patients with high TMB or non-squamous harboring SMARCA4 mutations enjoyed longer PFS in the toripalimab arm. Because the survival curves of these populations were not depicted, we only performed the analysis for patients with different histologies (squamous and non-squamous). Therefore, it was important to conduct cost-effectiveness analysis among specific subgroups to make the optimal decision for a specific population.

In conclusion, the current study evaluated the economic value of toripalimab plus chemotherapy for NSCLC patients with different histologies. Positive results were drawn that combined therapy overwhelmed chemotherapy alone in the ITT, squamous, and non-squamous NSCLC populations.

## Data Availability

The original contributions presented in the study are included in the article/Supplementary Material; further inquiries can be directed to the corresponding author.

## Supplementary material

SUPPLEMENTARY FIGURE S1 Cost-effectiveness acceptability curves **(A)**: Intention-to-treat population, **(B)**: patients with squamous NSCLC, and **(C)**: patients with non-squamous NSCLC. WTP, willingness to pay; T+chemo, toripalimab plus chemotherapy.
