# Supplementary figures and images for: Cost-effectiveness analysis of toripalimab plus chemotherapy as the first-line treatment in patients with advanced non-small cell lung cancer (NSCLC) without EGFR or ALK driver mutations from the Chinese perspective
Source: Front Pharmacol. 2023 May 17;14:1133085. doi: 10.3389/fphar.2023.1133085 (PMC10229795; doi:10.3389/fphar.2023.1133085)

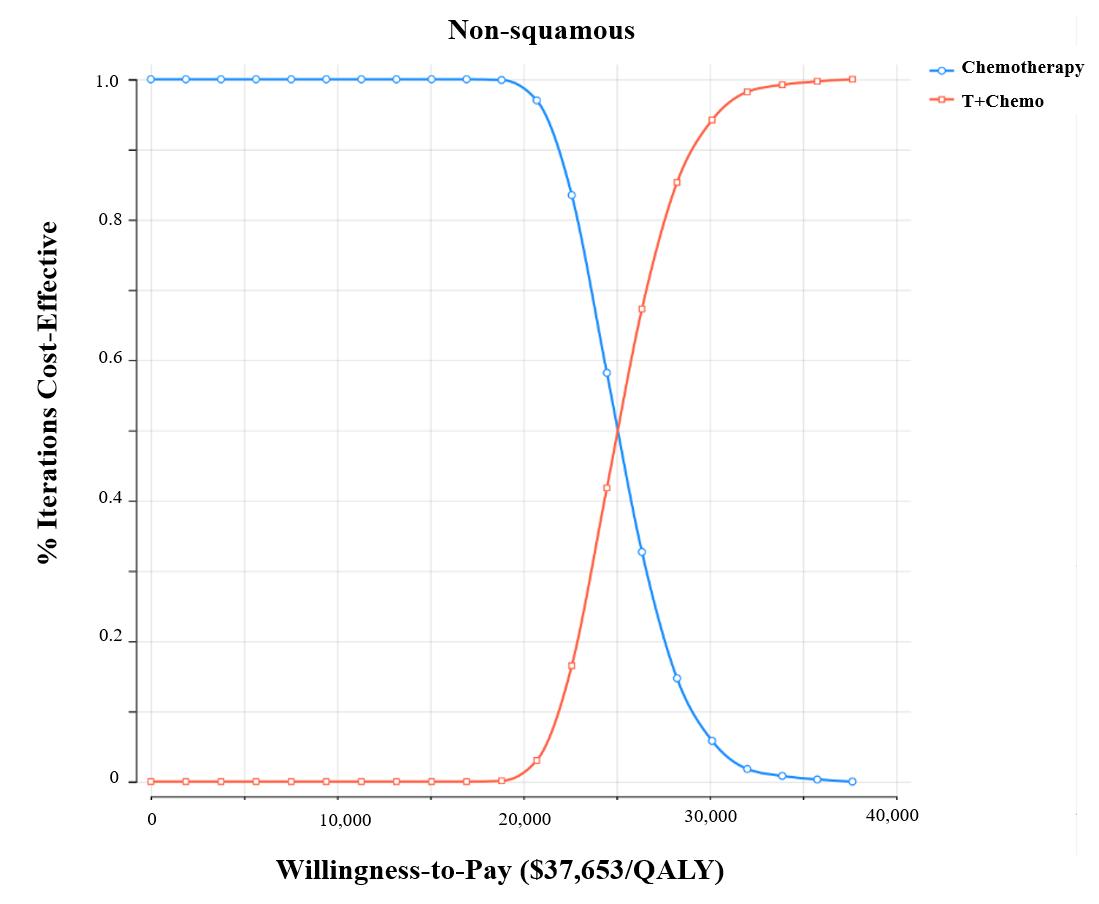

Supplement: Supplementary file 1 [file Image3.JPEG]

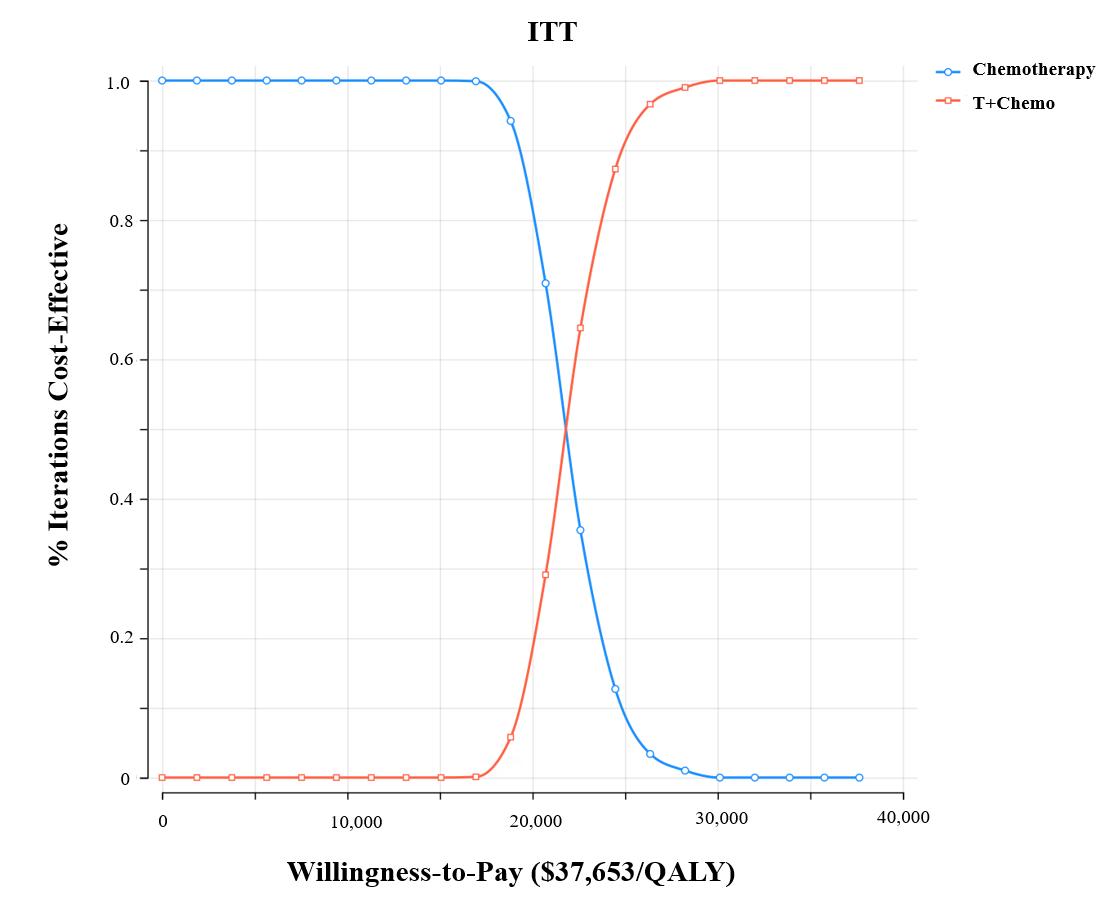

Supplement: Supplementary file 2 [file Image1.JPEG]

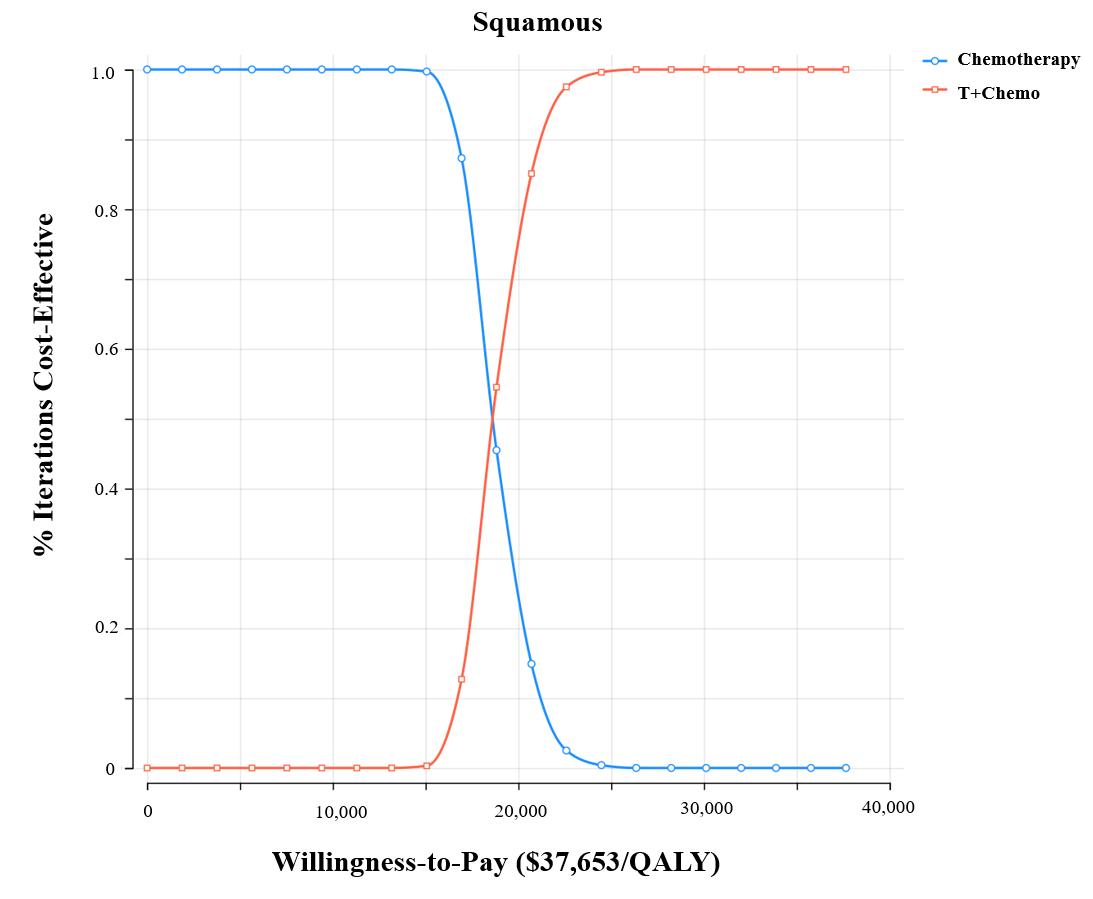

Supplement: Supplementary file 3 [file Image2.JPEG]
